# The role of feedback in visual masking and visual processing

**DOI:** 10.2478/v10053-008-0020-5

**Published:** 2008-07-15

**Authors:** Stephen L. Macknik, Susana Martinez-Conde

**Affiliations:** Barrow Neurological Institute, Phoenix, USA

**Keywords:** visual, masking, feedback, humans, monkeys, metacontrast, paracontrast, electrophysiology, optical imaging, fMRI, psychophysics, vision, awareness, attention, consciousness, standing wave

## Abstract

This paper reviews the potential role of feedback in visual masking, for and against. Our analysis reveals constraints for feedback mecha- nisms that limit their potential role in visual masking, and in all other general brain functions. We propose a feedforward model of visual masking, and provide a hypothesis to explain the role of feedback in visual masking and visual processing in general. We review the anato-my and physiology of feedback mechanisms, and propose that the massive ratio of feedback versus feedforward connections in the visual system may be explained solely by the critical need for top-down attentional modulation. We discuss the merits of visual masking as a tool to discover the neural correlates of consciousness, especially as compared to other popular illusions, such as binocular rivalry. Finally, we propose a new set of neurophysiological standards needed to establish whether any given neuron or brain circuit may be the neural substrate of awareness.

## AN INTRODUCTION TO VISUAL MASKING

**Figure 1.**
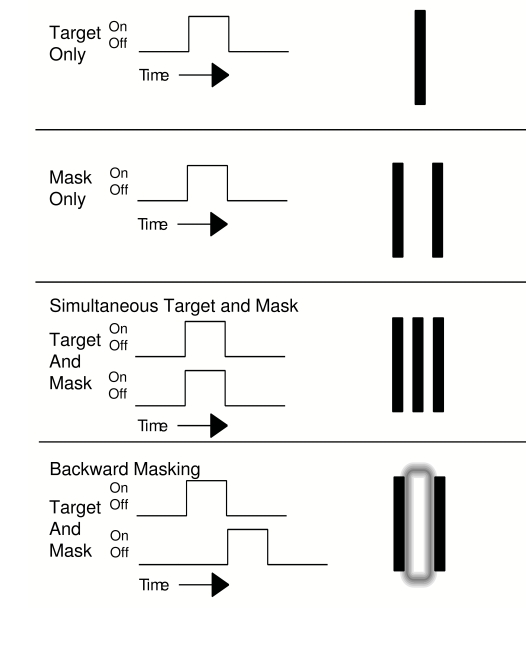
Perception of a target and mask with respect to temporal arrangement. Reprinted from Macknik (2006).

Visual masking illusions come in different flavors, but in all of them a visual stimulus, or some specific aspect of that stimulus (for instance the semantic content of a visually displayed word) is rendered invisible (or less visible) by modifying the context in which the stimulus is presented. Thus visibility is reduced without modifying the physical properties of the stimulus itself. Visual masking illusions allow us to examine the brain’s response to the same physical target under varying levels of visibility. These remarkable illusions may allow us to discover many, if not all, of the minimal set of neural conditions that cause visibility, by simply measuring the perceptual and physiological effects of the target when it is visible versus invisible during visual masking. See Figure 1 for a description of a type of visual masking called metacontrast masking, or backward masking, in which the target that is rendered invisible is presented before the mask.

Visual masking was discovered almost 140 years ago (Exner, 1868). We and others have shown that the neural correlate of backward masking is the suppression of the target’s after-discharge (Macknik & Livingstone, 1998; Macknik & Martinez-Conde, 2004b). Forward masking, in which the target is rendered invisible by a preceding mask, is correlated to the suppression of the target’s onset-response (Judge, Wurtz, & Richmond, 1980; Macknik & Livingstone, 1998; Schiller, 1968). The suppressive action of masking takes place at the spatiotemporal edges of the target, and it is driven by the spatiotemporal edges of the mask (Macknik, 2006; Macknik, Martinez-Conde, & Haglund, 2000). Together, these results suggest that stimulus visibility is caused by the transient bursts of neural activity that occur at the spatiotemporal edges of stimuli: when these bursts are inhibited by the action of a mask, visibility is reduced. We have proposed that all of the seemingly complex timing actions of visual masking are explained by one of the simplest neural circuits in the brain: lateral inhibition (Macknik, 2006; Macknik & Livingstone, 1998; Macknik & Martinez-Conde, 2004b; Macknik et al., 2000). Other studies have also proposed that lateral inhibition may explain visual masking effects (Bridgeman, 1971; Francis, 1997; Herzog, Ernst, Etzold, & Eurich, 2003; Weisstein, 1968; Weisstein, Ozog, & Szoc, 1975). However these other models have not explicitly captured or explained the role of the after-discharge in visibility and backward masking.

Bridgeman recorded from neurons in monkey striate cortex and concluded that early components of the target response were unaffected during backward masking, whereas late components were suppressed (Bridgeman, 1980). However, late components were defined as the average firing for a 210-310 ms period that started 70 ms after the onset of the mask (irrespective of target onset), and so it was not possible to determine whether the effects seen were relevant to target responses, mask responses, or both. Furthermore, this study did not employ automatic eye position monitoring (an assistant viewed the monkey’s face on a TV screen to determine if eye movements occurred), and thus it was not possible to know the relationship (or lack thereof) between the receptive field and the position of the target or mask. Also, Bridgeman did not vary the duration of the target or mask, and so could not have differentiated between onset-response and after-discharges. Finally, Bridgeman concluded that late components in the neural responses were caused by a combination of cortical reverberations [predicted by his lateral inhibitory model (Bridgeman, 1971)], and “cognitive influences”, which are presumably a function of feedback processes. However, neither Bridgeman’s, nor other physiological studies of visual masking, have identified such reverberatory activity. Our lateral inhibition model thus varies significantly from Bridgeman’s in that we have proposed that both onset-responses and after-discharges are due to the target’s temporal edges and that visual masking is a function of feedforward (non-reverberatory) lateral inhibitory interactions between target and mask.

Some groups have argued that lateral inhibition may not be the main circuit underlying visual masking, because it is too low-level to explain high-level masking effects such as object-substitution masking, feature integration, and the role of attention (Enns, 2002). However, we and others have proposed that lateral inhibition circuits that lie in high-level visual areas should indeed have high-level cognitive effects (Bridgeman, 2006; Francis & Herzog, 2004; Herzog et al., 2003; Macknik, 2006; Macknik & Martinez-Conde, 2004b). Nevertheless, the fact that lateral inhibition can explain visual masking does not itself rule out that other circuits, such as feedback inputs, may also be involved (Breitmeyer & Öğmen, 2006; Enns & Di Lollo, 1997; Haynes, Driver, & Rees, 2005; Lamme, Zipser, & Spekreijse, 2002; Thompson & Schall, 1999). Here we analyze the potential strengths and weaknesses of the various proposed feedback models of visual masking.

## Arguments for feedback in visual masking

### Öğmen and Breitmeyer’s two-channel theory of visual masking

**Figure 2.**
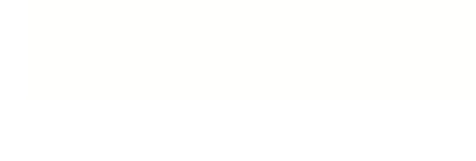
(**A**) The sequence of events during the course of a visual masking psychophysics trial. The trial started with a delay of 500 to 1500 msec. In backward masking conditions, the target was presented, followed by the mask. In forward masking conditions, masks came before targets. After termination of the second stimulus (mask or target) there was another 500 msec delay, after which the subject indicated which side had the longer target. (**B**) A schematic view of the various timing parameters used. SOA = Stimulus Onset Asynchrony, the interval between the onset of target and of mask; STA = Stimulus Termination Asynchrony, the interval between termination of target and of mask; ISI = Inter-Stimulus Interval, between the termination of the target and the onset of the mask (backward masking) or between the termination of the mask and the onset of the target (forward masking). Reprinted from Macknik & Livingstone (1998).

**Figure 3.**
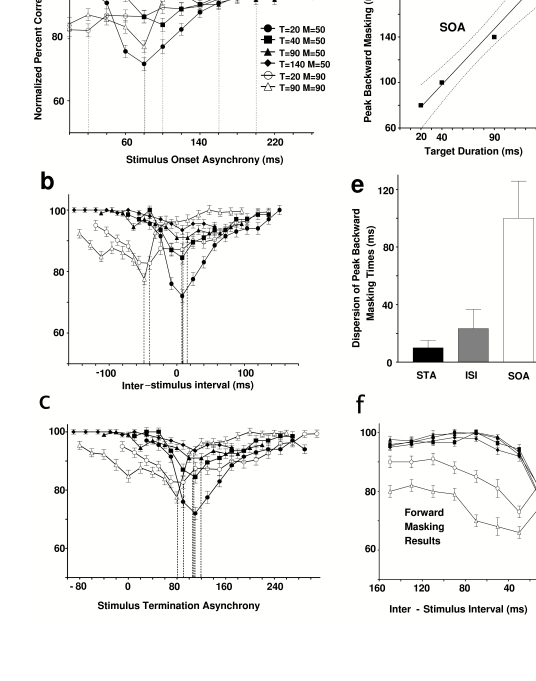
Psychophysical measurements of the timing parameters important for visual masking. “T” represents the duration (in milliseconds) of the target and “M” represents the duration of the mask. Results represent average for 25 subjects. (**A**) Results from backward masking conditions plotted on a stimulus onset asynchrony (SOA) scale. Note that the points of peak masking (the x-intercepts of the drop-lines) are widely dispersed. (**B**) Results from panel A replotted here as a function of inter-stimulus interval (ISI). The points of peak masking tend to cluster in two places, correlated with mask duration (open symbols vs. closed symbols). (**C**) Results from panel A replotted here on a stimulus termination asynchrony (STA) scale. The points of maximal masking are no longer dispersed, and instead cluster around an STA of about 100 ms +/- 20 ms. (**D**) Linear regression (with 95% confidence intervals) of peak backward masking times in terms of SOA when the mask was 50 ms in duration. (**E**) The amount of dispersion of peak backward masking times for data tested on a scale of stimulus termination asynchrony (STA), inter-stimulus interval (ISI), and stimulus onset asynchrony (SOA). Notice that the peak backward masking times are least dispersed on an STA scale. Thus STA is the best predictor of backward masking. (**F**) Results from forward masking conditions; the optimal predictor of peak masking is the ISI between the termination of the mask and the onset of the target. Reprinted from Macknik & Livingstone (1998).

**Figure 4.**
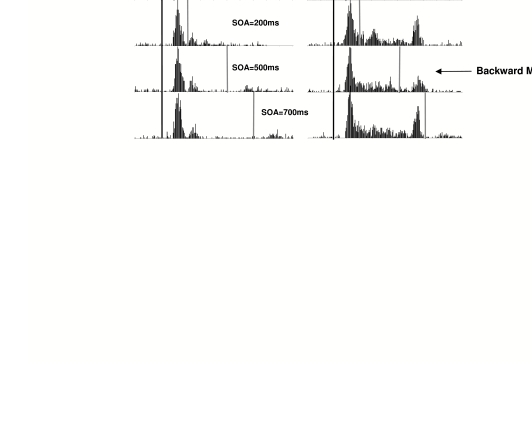
Multi-unit recording from upper layers of area V1 in an anesthetized rhesus monkey. The aggregate receptive field was foveal, 0.1° square, and well-oriented. In contrast to the recordings from alert animals, where eye movements occur frequently, the mask was largely outside the receptive field. The vertical bars (gray for mask, black for target), indicate the onset time of the stimuli. Notice that under conditions that best correlate with human forward masking (ISI = 0 ms, here corresponding to SOA = -100 ms) the main effect of the mask is to inhibit the transient onset-response to the target. Similarly, in the condition that produces maximum backward masking in humans (STA = 100 ms; here corresponding to SOA = 100 ms for the 100 ms stimulus on the left, SOA = 500 for the 500 ms stimulus on the right), the after-discharge is specifically inhibited. Each histogram is an average of 50 trials with a bin width of 5ms. Modified from Macknik & Livingstone (1998).

In this volume of Advances in Cognitive Psychology, Breitmeyer presents the latest version of the famous two-channel model of visual masking, which includes a requirement for feedback circuits (Breitmeyer, 2006). Breitmeyer and Ganz’s (Breitmeyer & Ganz, 1976) original version of the two-channel model of masking proposed that there were two different visual information channels, one exhibiting fast and transient characteristics (so that information traveled quickly through the channel) and one exhibiting slow and sustained characteristics. The idea was that, during backward masking, the neural representation of the mask would travel rapidly through the transient channel and thus intercept the sustained channel’s neural representation of the target in cortical circuits where the two channels meet. The fast representation of the mask would thus suppress the slow representation of the target, decreasing target visibility. The difference in latency (in the sense of propagation speed) between the two channels was modeled as a fixed physiological parameter. Thus the two-channel model required that the target and mask be presented with a specific Stimulus Onset Asynchrony (SOA, see Figure 2). Macknik and Livingstone (1998), and Macknik and Martinez-Conde (2004a) probed this “transient-on-sustained inhibition” hypothesis psychophysically by testing whether backward masking occurred at a specific SOA, or not. They found that the timing of masking was not determined by SOA but it depended on a previously untested temporal characteristic, Stimulus Termination Asynchrony (STA, see Figure 2). Figure 3 shows that STA determines the perceptual timing of backward masking more accurately than either SOA or Inter-Stimulus Interval (ISI). Thus the transient-on-sustained inhibition hypothesis of backward masking is not sustainable on psychophysical grounds. Macknik and Livingstone (1998) also showed that forward masking was better explained by ISI than by either SOA or STA. Macknik and Livingstone further tested the neurophysiological underpinnings of visual masking by recording the neural activity from single units in monkey primary visual cortex (V1) during forward and backward visual masking. The results confirmed previous physiological findings (Judge et al., 1980; Schiller, 1968) that the neural correlate of forward masking was the suppression of the target’s onset-response. They also showed that backward masking was correlated to the suppression of the target’s after-discharge (Figure 4). This physiological finding correlated precisely to the psychophysics. It also explained why STA was the best timing parameter to describe peak backward masking: because backward masking occurs when the target’s after-discharge is suppressed by the mask, it follows that if either the target or the mask varies in duration, the relative temporal delay between the termination of the target and mask should be critical.

**Figure 5.**
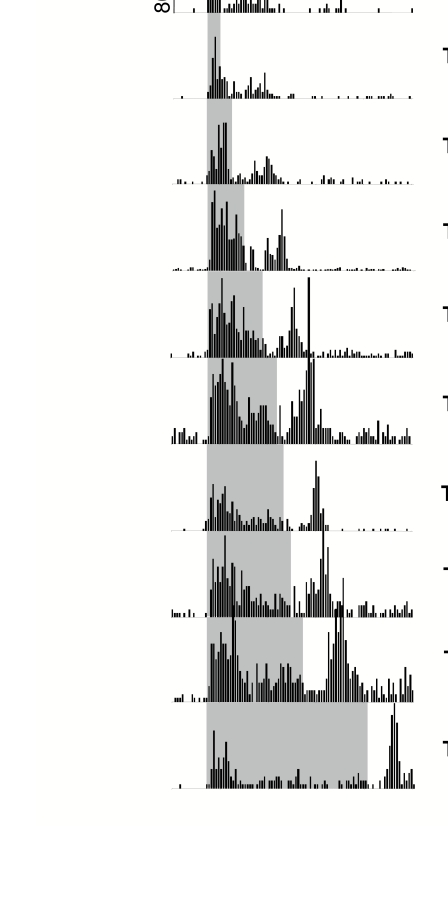
Recording from a typical single neuron from monkey area V1 that was stimulated with a target of various durations. The magnitude of the after-discharge grows as the target duration increases. Reprinted from Macknik & Martinez-Conde (2004a).

Breitmeyer and Öğmen (2006) revised the two-channel model, now called the retino-cortical dynamics (RECOD) model. One motivation for revision was provided by Super, Spekreijse, and Lamme (2001), who suggested that the late responses of V1 neurons, such as the after-discharges in Macknik and Livingstone (1998), were caused by feedback from higher visual areas, rather than from the stimulus’s termination. Breitmeyer and Öğmen (2006) thus proposed that the two channel hypothesis was essentially correct, if one considered that the fast and slow channels were not the magnocellular and parvocellular retino-geniculocortical pathways, as previously modeled, but were instead feedforward ascending input (fast channel) and feedback from higher visual areas (slow channel). In the recast two-channel model, the feedforward input from the mask would suppress the (delayed) feedback input from the target (i.e. the after-discharges), thus causing suppression of the target’s visibility. One problem with this idea, however, is that after-discharge timing varies as a function of stimulus termination time (Figure 5). This indicates that after-discharges are not caused by feedback from the stimulus’s onset. If after-discharges were caused by feedback, the areas providing the feedback would need to be able to predict the moment of termination of the stimulus. To the best of our knowledge, no study previous to Macknik and Livingstone (1998) varied the duration of both targets and masks to assess the role of after-discharges in visual masking. Thus it had not been possible to differentiate between the role of feedforward and feedback circuits in the formation of after-discharges.

In summary, the RECOD model, which is dependent on the idea that after-discharges are due to feedback and relies on SOA as the primary timing parameter, is not supported by the available physiological and psychophysical data.

### Lamme’s recurrent feedback hypothesis of visual awareness and masking

Lamme’s model of visual awareness and masking, based on physiological recordings in the awake monkey, suggests that onset-responses are due to feedforward input, and late responses (i.e. after-discharges) are due to recurrent feedback (Lamme et al., 2002). Lamme’s model superficially agrees with our lateral inhibition feedforward model in that backward masking is correlated to the suppression of late responses. But a key difference between the two models is that, in Lamme’s model, the suppression of late responses is caused by a decrease in feedback from higher visual areas, whereas in our model late responses are suppressed by direct feedforward lateral inhibition. In Lamme’s model, the effect of masking should be stable with respect to SOA. That is, target duration should be irrelevant because late responses are proposed to occur as a function of feedback, which is itself generated by the target’s onset-response as it rises through the visual hierarchy. In our model, target duration is a critical parameter, because after-discharges are feedforward transients caused by target termination. Because masking strength does vary as a function of target duration (Macknik & Livingstone, 1998), Lamme’s feedback model can be ruled out on psychophysical grounds. Rossi, Desimone and Ungerleider (2001) have moreover demonstrated that the results reported by Lamme’s group (Lamme, 1995; Lee, Mumford, Romero, & Lamme, 1998; Zipser, Lamme, & Schiller, 1996), that monkey V1 neurons segregate figure from ground, may have been caused by receptive field position changes due to uncontrolled eye movements (i.e. the receptive field physically traveled over the border from the figure to the background).

In spite of these arguments, Lamme’s group has maintained that late responses are due to feedback: Their 1997 Association for Research in Vision and Ophthalmology conference abstract described that the surgical removal of the entire extrastriate visual cortex of a monkey (V3, V3a, V4, V4t, MT, MST, FST, PM, DP, and 7a) led to a reduction of area V1 late responses (Lamme, Zipser, & Spekereijse, 1997). However, surgical ablations are irreversible by definition, and the nature of the technique is such that it often leads to inconclusive results. The surgical removal of the extrastriate cortex in a monkey involves the resection of a large portion of the entire cerebral cortex, and thus causes massive traumatic damage to the brain as a result, including substantial damage to the cortical lymphatic and vascular systems. Therefore it is unclear exactly what processes may or may not be affected by such a drastic ablation. A less complicated test of the late response’s origin is to vary the duration of the target, which establishes whether the late response timing varies as a function of target duration (and is thus a feedforward after-discharge), or not (Macknik & Livingstone, 1998; Macknik & Martinez-Conde, 2004b; Macknik et al., 2000). Lamme and colleagues did not conduct such a test, and no other physiological studies that we know of have supported their claim that late responses are caused by feedback. Thus the more parsimonious explanation is that late responses are feedforward after-discharges that occur at the termination of the stimulus.

Most cortical visual neurons are complex in nature (they receive inputs from both on and off channels). Thus every complex cell that responds to a given stimulus should produce an after-discharge when that stimulus is extinguished. Therefore any model that proposes that after-discharges are due to feedback, and not to feedforward inputs, must also explain why expected feedforward after-discharges are otherwise missing, only to be replaced by feedback. No such model has been forthcoming.

### Object substitution masking

Object substitution masking (OSM) (Enns & Di Lollo, 1997) is an effect in which a target object is suppressed by a mask of similar shape, even though the mask does not abut the target spatially (as it is necessary in other types of masking discussed here). Enns and Di Lollo proposed that OSM must be caused by high-level feedback to early visual cortex:

1) The strength of OSM is modulated greatly by covert voluntary attention. This suggests that the masking circuits are co-localized with, or affected by, high-level cognitive circuits.

2) We and others have shown that some types of visual masking are processed within early visual areas (Macknik & Haglund, 1999; Macknik & Livingstone, 1998; Macknik & Martinez-Conde, 2004a; Macknik et al., 2000; Tse, Martinez-Conde, Schlegel, & Macknik, 2005). Enns (2002) proposed that these early visual areas must receive input from high-level areas to process visual masking.

3) The OSM effect is based on specific object shapes. Since object shape is processed within higher extrastriate visual areas (Kobatake & Tanaka, 1994; Tanaka, Sugita, Moriya, & Saito, 1993; Wang, Tanaka, & Tanifuji, 1996), the circuits that process visual masking must be co-localized with higher visual areas and then feedback to early visual areas (as in 2, above).

**Figure 6.**
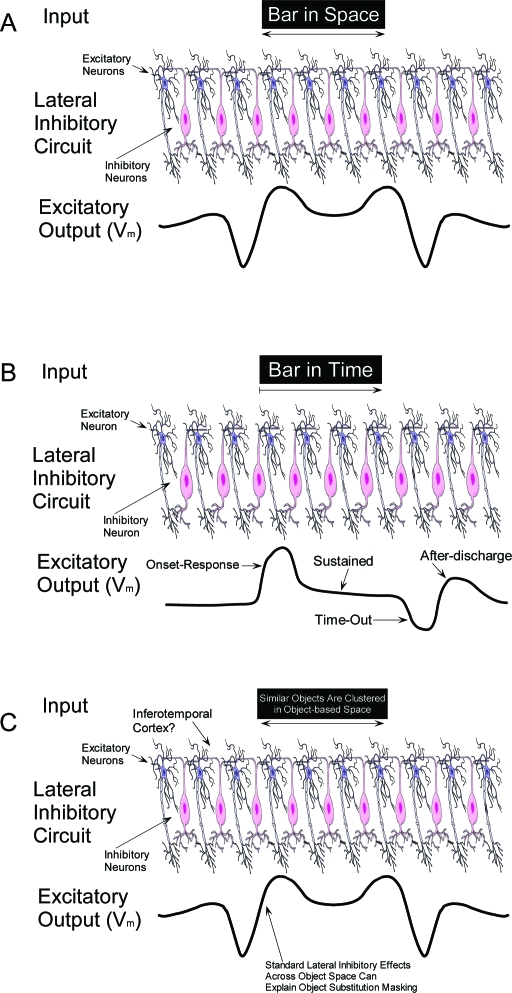
(**A**) A representation of the spatial lateral inhibition model originally proposed by Hartline and Ratliff (Ratliff, 1961; Ratliff et al., 1974). The excitatory neurons in the center of the upper row receive excitatory input from a visual stimulus. This excitation is transmitted laterally in the form of inhibition, resulting in edge enhancement of the stimulus: the neuronal underpinnings of the Mach Band illusion (Mach, 1965). (**B**) One excitatory and one inhibitory neuron taken from the spatial model in panel A, now followed through an arbitrary period of time. Several response phases are predicted, including the onset- response, and the transient after-discharge (Adrian & Matthews, 1927). (**C**) A representation of the lateral inhibition model interactions within object space. The excitatory neurons in the center of the upper row receive excitatory input from a visual stimulus (for instance an object or group of objects with similar shapes). This excitation is transmitted laterally in the form of inhibition, resulting in “edge enhancement” across object space, equivalent to the retinotopic edge enhancement in earlier levels of the visual pathway (i.e. panel A). These interactions may lead to object-based visual masking illusions. Therefore low-level lateral inhibition may explain object substitution masking (OSM).

Despite these seemingly high-level interactions, we have proposed that OSM may be explained by feedforward lateral inhibition circuits (Macknik, 2006; Macknik & Martinez-Conde, 2004a, 2004b). Lateral inhibition is a ubiquitous brain circuit, thus it does not only exist within early visual areas, but also within the high-level visual areas that process object shape (such as the inferotemporal cortex; IT). Lateral inhibition circuits within high-level areas may thus cause complex perceptual results. Let us first consider how lateral inhibition may work, across both retinotopic space and time, to cause low-level visual masking. Figure 6a represents the spatial lateral inhibition model originally proposed by Hartline and Ratliff (Ratliff, 1961; Ratliff, Knight, Dodge, & Hartline, 1974). Here, the excitatory neurons in the center of the upper row receive excitatory input from a visual stimulus (a bar of light, for instance). This excitation is then transmitted laterally in the form of inhibition, resulting in edge enhancement of the stimulus: the neuronal underpinnings of the Mach band illusion (Mach, 1965). One can easily imagine how the spatial edges of the mask may potentially nullify the responses caused by the edges of the target, if the mask’s edges are positioned spatially so as to inhibit the target’s edge enhancement. One might expect that the target may in turn also inhibit the mask (which does happen to some extent), but if we consider the temporal aspects of the model it becomes clear why this inhibitory interaction is largely from mask to target. Let us now look at the same network through time: Figure 6b shows one excitatory and one inhibitory neuron from the spatial network in Figure 6a, followed through an arbitrary period of time. Several temporal phases of response occur as a function of the lateral inhibitory network, thus explaining the formation of the onset-response, sustained period, and the transient after-discharge (Macknik & Martinez-Conde, 2004b). The temporal effects of lateral inhibition thus explain the seemingly mysterious timing of target and mask in visual masking: the mask’s onset response and after-discharge must temporally overlap (and spatially overlap, as described above) the target’s onset response and/or after-discharge, in order to suppress the perception of the target.

If we now assume that this same simple circuit is embedded within a high-level visual area, such as the inferotemporal cortex (IT), we will see that its biophysical behavior remains fundamentally the same. However, its significance to perception may now be extended to the interactions between whole objects (regardless of their location in retinotopic space), rather than being constrained to the interactions between edges across retinotopic space, Figure 6c. This simple hypothesis may explain why OSM is strongest when the mask is similar in shape to the target (i.e. because shape similarity will make the target and mask lie close to each other in the object-based topographical cortical map). It also explains why the target and mask need not be near each other retinotopically during OSM.

One important facet of OSM is the role of attention. Several groups have hypothesized that OSM must be mediated by high-level circuits because it is strongly modulated by attentional load (Bridgeman, 2006; Enns & Di Lollo, 2000), whereas low-level forms of masking are modulated much less by attention. However, the role of attention in OSM may be a red herring, at least to the study of visual masking. Attention may be mediated by a separate dissociated mechanism all its own: this system may then affect circuits that mediate visual masking, just as it affects other visual processes (i.e. motion perception, shape perception, cognition, awareness, etc). The fact that attention plays a stronger role in OSM than in simpler forms of masking strengthens the lateral inhibition model of OSM: Because high-level visual areas are modulated more strongly by attention than are low-level visual areas, it makes sense that the lateral inhibition circuits responsible for OSM may be more strongly modulated by attention than the lateral inhibition circuits responsible for simpler forms of visual masking within lower visual areas.

### Coupled interactions between V1 and fusiform gyrus

Haynes, Driver and Rees (2005) proposed that target visibility derives from the coupling of area V1 BOLD activity with fusiform gyrus BOLD activity. This hypothesis suggests a feedback pathway from the fusiform gyrus to V1, which would then mediate the functional coupling. However, V1 activation in this study may not be related to target visibility, but rather may indicate an experimental confound with top-down attention (Macknik, 2006). Subjects were required to attend actively to the target: focused covert attention causes increased BOLD activity in human V1 (Brefczynski & DeYoe, 1999). Haynes, Driver and Rees attempted to control for this attentional confound by including a condition in which the subject’s attention was directed away from the target. However, in the final analysis in which coupling was found, the target-unattended condition data was not included, and so the attentional confound cannot be ruled out. Thus the result may be due to the attentional aspect of the attended condition, and not to visual masking per se.

### Frontal lobe processing of visual masking

Thompson and Schall recorded from single-units in the frontal lobes of the awake monkey and concluded that visual masking cannot be processed in the early visual system, but is instead processed in the frontal eye-fields (FEF) (Thompson & Schall, 1999; Thompson & Schall, 2000). They suggested that the neural correlate of visual masking is the “merging” of target and mask responses, rather than the inhibition of target responses. However, their target was almost 300 times dimmer than their mask, and so target and mask responses may have merged because of the different response latencies one would expect from a dim and a bright stimulus (Albrecht & Hamilton, 1982; Gawne, Kjaer, Hertz, & Richmond, 1996). Moreover, the SOAs used were approximately equivalent to the difference in latencies that would be expected from a 300X luminance difference. Because of this combined SOA and latency confound, the authors could not have differentiated whether the target’s response was inhibited by the mask, or whether the mask’s larger response occluded the small and delayed dim-target response. In previous experiments by us and others (Macknik & Haglund, 1999; Macknik & Livingstone, 1998; Macknik & Martinez-Conde, 2004a, 2004b; Macknik et al., 2000; Tse et al., 2005), target and mask were of equal contrast to avoid the latency confound. Furthermore, when Thomson and Schall used either very long or short SOAs (in which the target and mask responses could be differentiated in time), they found that it was the *mask’s* response that was suppressed rather than the *target’s*; this is opposite to what one would expect in visual masking. Finally, the monkey’s task was to detect a blue target against a field of white distracter masks, and so it is possible that differential attentional effects would suppress the mask but not the target. These types of attentional effects have been documented in the FEF and other parts of the brain when the primate is trained to direct its attention to particular colored stimuli (i.e. the blue target) and ignore others (i.e. the white mask) (Bichot & Schall, 1999; Reynolds, Chelazzi, Luck, & Desimone, 1994; Reynolds, Chelazzi, & Desimone, 1999; Reynolds & Desimone, 1999; Reynolds, Pasternak, & Desimone, 2000). Thus Thompson and Schall’s data may be further confounded by the effects of selective attention, rather than being the direct result of visual masking.

## Arguments against feedback in visual masking

### Feedback in visual masking

To summarize the previous sections, there are several facts to consider about the role of feedback in visual masking:

1) The neural correlate of forward masking is the inhibition of the target’s onset response (Macknik & Livingstone, 1998).

2) The neural correlate of backward masking is the inhibition of the target’s after-discharge (Macknik & Livingstone, 1998).

3) The after-discharge occurs as a function of stimulus termination. Responses that occur as a function of stimulus termination cannot be due to feedback processes. Therefore, after-discharges are the result of feedforward connections (Macknik & Livingstone, 1998; Macknik & Martinez-Conde, 2004a, 2004b; Macknik et al., 2000).

a) It follows that the timing of any response due to feedback should be invariant with respect to stimulus duration. Since visual masking timing varies as a function of target duration, visual masking is not due to feedback (Macknik & Livingstone, 1998; Macknik & Martinez-Conde, 2004a, 2004b; Macknik et al., 2000; Tse et al., 2005).

4) The relative duration and timing of target and mask determine the timing and neural correlates of forward and backward masking (Macknik & Livingstone, 1998; Macknik & Martinez-Conde, 2004b; Macknik et al., 2000).

**Figure 7.**
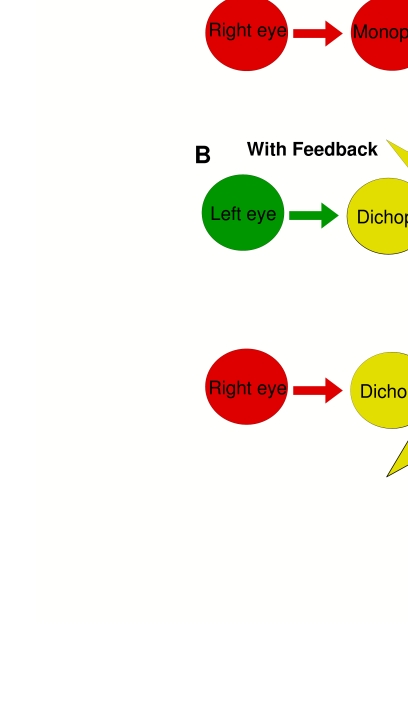
Overriding issues when considering the viability of feedback mechanisms. (**A**) A general model of early visual binocular integration without invoking feedback mechanisms. (**B**) If significant feedback existed between the initial dichoptic levels of processing and earlier monoptic levels, the earlier levels should behave in the same way as the dichoptic levels (i.e. they would become dichoptic by virtue of the feedback). Reprinted from Macknik (2006).

The above facts argue against a model of visual masking in which feedback plays a critical role. Nevertheless, the research discussed thus far has not directly tested the potential role of feedback. This section will describe experiments we have carried out to measure the strength of feedback in visual masking (Macknik & Martinez-Conde, 2004a, 2004b; Tse et al., 2005). If feedback does play a role in visual masking, we should be able to test several strong predictions concerning the behavior of the neural circuits involved. For instance, Enns (2002), Breitmeyer and Öğmen (2006), and Lamme, Zipser and Spekreijse (2002) have proposed that low-level circuits exhibit masking only due to feedback from high-level circuits. If this hypothesis is correct, then low-level circuits should exhibit the types of masking produced by high-level circuits. Figure 7 outlines the logic of this argument for monocular visual circuits that receive feedback from binocular circuits capable of dichoptic masking. If the activity within early monoptic circuits correlates with the perception of visual masking due solely to feedback from dichoptic circuits [as argued by Enns (2002) ], it follows that the activity in early monoptic circuits must also correlate with the perception of dichoptic masking.

### The perception of monoptic and dichoptic visual masking

**Figure 8.**
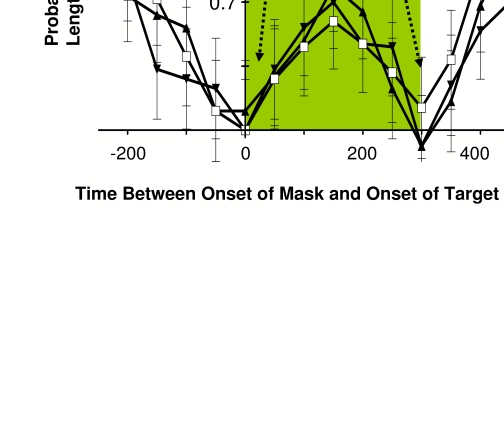
Psychophysical examination of dichoptic versus monoptic masking in humans. Human psychophysical measurements of visual masking when 10 ms duration target and 300 ms duration mask were presented to both eyes together (monoptic masking) and to the two eyes separately (dichoptic masking). The probability of discriminating correctly the length of two targets is diminished, in the average responses from 7 subjects, when targets were presented near the times of mask onset and termination. This is true regardless of whether the target and mask were presented to both eyes (open squares), or if the target was presented to one eye only and the mask was presented to the other (target = left, mask = right: closed upright triangles; target = right, mask = left: closed upside-down triangles). Open squares signify when the target was displayed with both shutters closed, showing that the stimuli were not visible through the shutters. When the mask and the target were presented simultaneously, both eyes’ shutters were necessarily open (dichoptic presentations using shutters are impossible when both stimuli are presented at the same time), and so between times 0-250 ms all four conditions were equivalent. Dichoptic masking is nevertheless evident when the target was presented before the mask’s onset (-250 to -50 ms on the abscissa), as well as when the target was presented after the mask had been terminated (300 ms to 500 ms on the abscissa). Reprinted from Macknik & Martinez-Conde (2004b).

The existence of “dichoptic” visual masking is one of the main reasons visual masking has been considered a cortical process (Harris & Willis, 2001; Kolers & Rosner, 1960; McFadden & Gummerman, 1973; McKee, Bravo, Smallman, & Legge, 1995; McKee, Bravo, Taylor, & Legge, 1994; Olson & Boynton, 1984; Weisstein, 1971). However, just because dichoptic masking must arise from binocular cortical circuits, does not mean that monoptic masking may not arise from monocular subcortical circuits (Macknik, 2006; Macknik & Martinez-Conde, 2004a). To be clear about the jargon: “monocular” means “with respect to a single eye”, and “monoptic” means either “monocular” or, “not different between the two eyes”. “Binocular” means “with respect to both eyes” and “dichoptic” means “different in the two eyes”. Thus, in dichoptic visual masking, the target is presented to one eye and the mask to the other eye, and the target is nevertheless suppressed. Excitatory binocular processing within the geniculocortical pathway occurs first in the primary visual cortex (Hubel, 1960; Le Gros Clark & Penman, 1934; Minkowski, 1920). Thus it has been assumed that dichoptic masking must originate from cortical circuits. The anatomical location in which dichoptic masking first begins is critical to our evaluation of most models of masking. It is also important to our understanding of LGN neurons and their relationship to the subcortical and cortical structures that feed-back onto them. In order to establish where dichoptic masking first begins, we first compared the perception of monoptic to dichoptic visual masking in humans over a wide range of timing conditions never before tested (Macknik & Martinez-Conde, 2004a), see Figure 8. We found that dichoptic masking was as robust as monoptic masking, and that it exhibited the same timing characteristics previously discovered for monoptic masking (Crawford, 1947; Macknik & Livingstone, 1998; Macknik et al., 2000).

The following experiments set out to measure the physiological correlates of monoptic and dichoptic visual masking in monkeys and humans.

### Monoptic and dichoptic visual masking in monkeys

**Figure 9.**
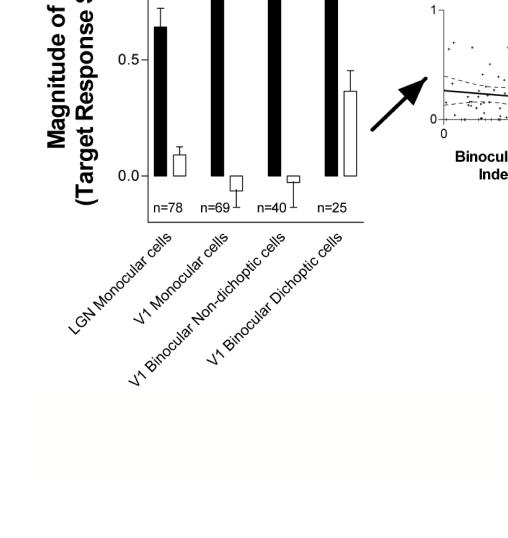
Summary statistics of monoptic vs. dichoptic masking responses in the LGN and area V1. Monoptic (black bars) and dichoptic (white bars) masking magnitude as a function of cell type: LGN, V1 monocular, V1 binocular (non-responsive to dichoptic masking), and V1 binocular (responsive to dichoptic masking) neurons. Inset shows the linear regression of dichoptic masking magnitude in V1 binocular neurons as a function of their degree of binocularity (all neurons plotted were significantly binocular as measured by their relative responses to monocular targets presented to the two eyes sequentially): BI of 0 indicates that the cells were monocular, while a BI of 1 means both eyes were equally dominant. Reprinted from Macknik & Martinez- Conde (2004b).

We recorded from LGN and V1 neurons in the awake monkey while presenting monoptic and dichoptic stimuli (Macknik & Martinez-Conde, 2004a). To the best of our knowledge, these were the first dichoptic masking experiments to be conducted with single-unit physiological methods. We found that monoptic masking occurred in all the LGN and V1 neurons we recorded from, whereas dichoptic masking occurred solely in a subset of V1 binocular neurons (Figure 9). We also discovered that, in V1 binocular neurons, excitatory responses to monocular targets were inhibited strongly by masks presented to the same eye, whereas interocular inhibition was surprisingly weak. We concluded that the circuits responsible for monoptic and dichoptic masking must exist independently in at least two brain levels, one in monocular circuits and one in binocular circuits. Furthermore, Enns (2002) proposed that early monoptic masking circuits exhibited masking due to feedback from dichoptic levels, which we did not find. If monoptic masking in early visual areas was the result of feedback from higher areas, then the feedback connections would also convey strong dichoptic masking from the later circuits. Thus the early circuits would inherit this trait with the feedback (Figure 7), and they would exhibit dichoptic masking as well as monoptic masking. Since the earlier levels do not exhibit dichoptic masking, we concluded that visual masking in monoptic regions is not due to feedback from dichoptic regions.

In summary, Macknik and Martinez-Conde (2004b) showed for the first time that dichoptic and monoptic masking are generated by two different circuits (i.e. one that lies in binocular cells and another that lies within monocular cells). Several studies have since verified this result psychophysically (Meese & Holmes, 2007; Petrov, Carandini, & McKee, 2005; Petrov & McKee, 2006). Therefore the above results support the parsimonious hypothesis that the main circuit underlying visual masking is lateral inhibition.

Figure 9 shows that the strength of monoptic masking increases, in an iterative fashion, with each successive stage of processing in the visual system. Correspondingly, Hubel and Wiesel (Hubel & Wiesel, 1961) found that inhibitory surrounds were stronger in the LGN than in the retina. We proposed that lateral inhibition mechanisms gather strength iteratively in successive stages of the visual hierarchy. The result that dichoptic inhibition is weak in area V1 may reflect such a general principle, given that V1 binocular neurons represent the first stage where dichoptic inhibition could exist in the ascending visual system. If our iterative inhibitory buildup hypothesis is correct, downstream binocular neurons in the visual hierarchy should show iteratively stronger interocular suppression and dichoptic masking. Further, dichoptic masking must become stronger downstream of V1, to account for the fact that the psychophysical magnitude of dichoptic masking is equivalent to that of monoptic masking (Figure 8).

### Monoptic and dichoptic visual masking in humans

To search for the neural correlates of masking at higher levels of the visual hierarchy, we turned to whole brain imaging (functional Magnetic Resonance Imaging; fMRI) techniques in humans (Tse et al., 2005). Masking illusions evoke reliable BOLD signals that correlate with perception within the human visual cortex (Dehaene et al., 2001; Haynes & Rees, 2005). Since the psychophysical strengths of monoptic and dichoptic masking are equivalent (Macknik & Martinez-Conde, 2004a; Schiller, 1965), we set out to find the point in the ascending visual hierarchy in which monoptic and dichoptic masking activity are both extant. This is the first point in the visual hierarchy at which awareness of visibility could potentially be maintained. Previous to this level, target responses will not be well inhibited during dichoptic masking: if these prior areas were sufficient to maintain visual awareness, the target would be perceptually visible during dichoptic masking conditions.

**Figure 10.**
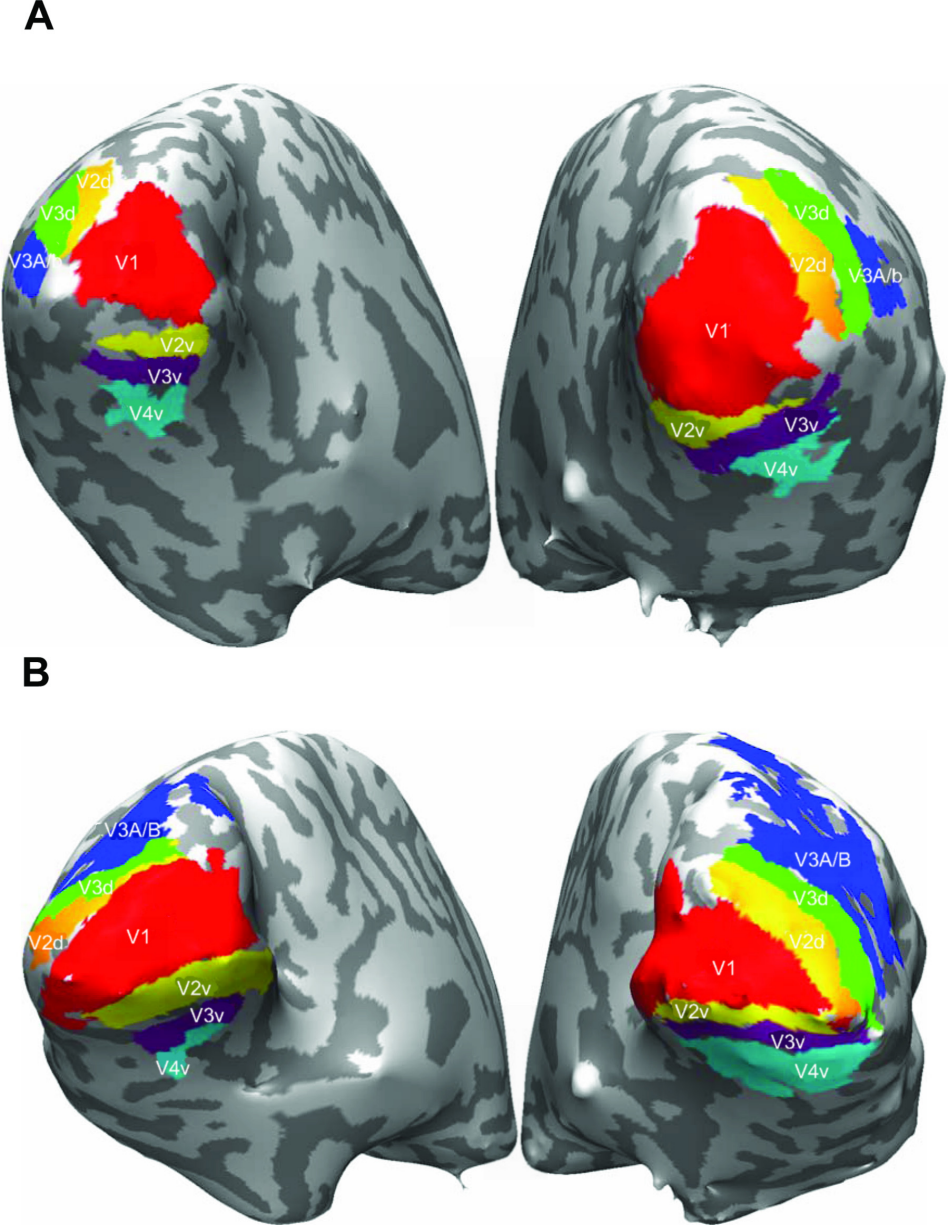
Examples of retinotopy mapping from two subjects. (A & B) Visual areas delineated by retinotopic mapping analysis are indicated in different colors. Reprinted from Tse, et al. (2005).

**Figure 11.**
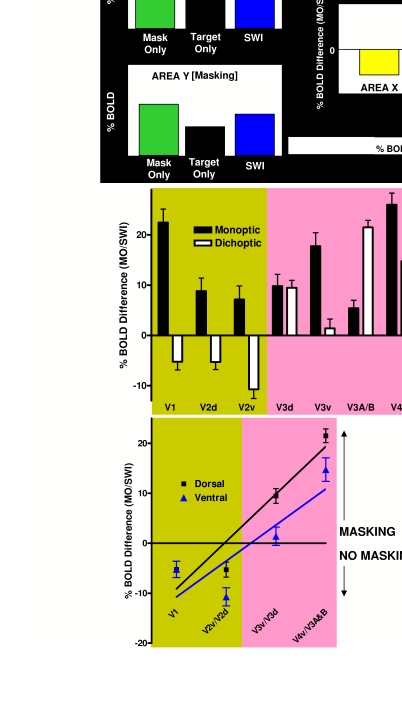
Retinotopic analysis of monoptic versus dichoptic masking. (**A**) The logic underlying the analysis of masking magnitude for hypothetical retinotopic areas. The Mask Only response is bigger than the Target Only response because masks subtend a larger retinotopic angle than targets, and are moreover presented twice in each cycle for 100 msec each flash, whereas the target is single-flashed for only 50 msec. If the target response adds to the mask response in the Standing Wave of Invisibility condition (SWI, see Figure 16) (because no masking percept was experienced), then the SWI response will be bigger than the Mask Only response. If the target does not add (masking percept), then the SWI response will be equal or smaller than the Mask Only response (as the mask itself may also be somewhat reciprocally inhibited by the target). (**B**) Monoptic and dichoptic masking magnitude (% BOLD difference of Mask Only / SWI conditions) as a function of occipital retinotopic brain area, following the analysis described in panel A. Negative values indicate increased activation to the SWI condition (no masking), whereas values ≥ 0 indicate unchanged or decreased SWI activation (masking). (**C**) Dichoptic masking magnitude (% BOLD difference of Mask Only / SWI conditions) as a function of occipital retinotopic brain area within the dorsal and ventral processing streams. The strength of dichoptic masking builds up throughout the visual hierarchy for both the dorsal (R2 = 0.90) and ventral (R2 = 0.72) processing streams. Reprinted from Tse, et al. (2005).

We measured BOLD signal in response to monoptic and dichoptic masking within individually mapped retinotopic areas in the human brain (Figure 10). Our results showed that dichoptic masking does not correlate with visual awareness in area V1, but begins only downstream of area V2, within areas V3, V3A/B, V4 and later (Figure 11). The results agreed with previous primate electrophysiological studies using visual masking and binocular rivalry stimuli (Logothetis, Leopold, & Sheinberg, 1996; Macknik & Martinez-Conde, 2004a; Sheinberg & Logothetis, 1997), as well as with one fMRI study of binocular rivalry in humans (Moutoussis, Keliris, Kourtzi, & Logothetis, 2005). We also found that the iterative increase in lateral inhibition we previously discovered from the LGN to V1 for monoptic masking (Figure 9), continued in the extrastriate cortex for dichoptic masking (Figure 11c). This is an important fact in localizing the circuits responsible for maintaining visibility and visual awareness. For instance, if the brain areas that maintained visual awareness exhibited only weak target suppression (i.e. as in early visual areas such as the LGN and V1), then target masking would be incomplete and targets would be perceptually visible during masking. Since the perception of dichoptic masking is as strong as that of monoptic masking, and since the neural activity evoked by the target is only weakly suppressed by dichoptic masks prior to area V3, it follows that the circuits responsible for visibility must lie in V3 or later, or else targets would not be perceptually suppressed during dichoptic masking.

**Figure 12.**
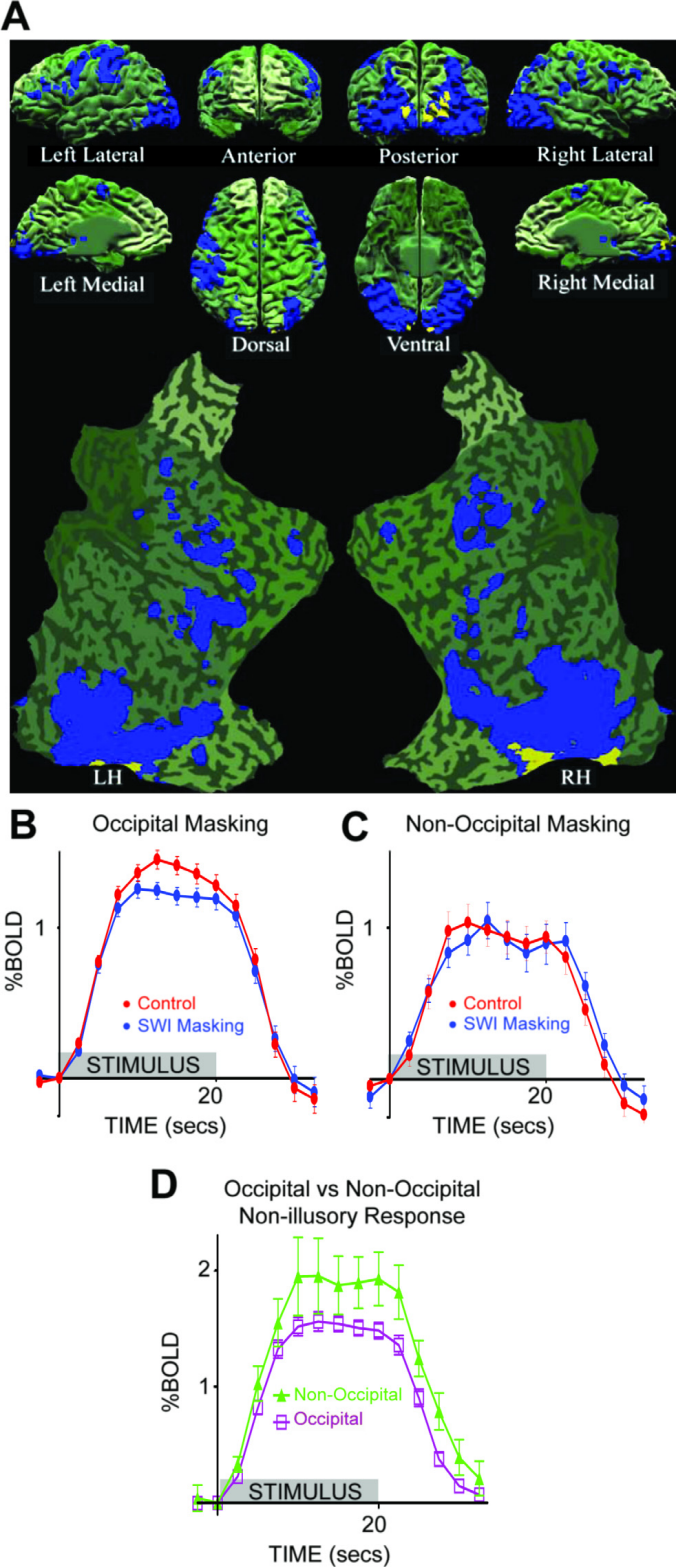
Localization of visibility-correlated responses to the occipital lobe. (**A**) An individual brain model from all perspectives, including both hemispheres flat-mapped, overlaid with the functional activation from 17 subjects. The green shaded areas are those portions of the brain that did not show significant activation to Target Only stimuli. The blue voxels exhibited significant target activation (Target Only activation > Mask Only activation). Yellow voxels represent a significant difference between Control (target and mask both presented, with target-visible) and SWI (target and mask both presented, with target-invisible) conditions, indicating potentially effective visual masking, and thus a correlation with perceived visibility. (**B**) Response time-course plots from Control versus SWI conditions in the occipital cortex. (**C**) Response time-course plots from Control versus SWI conditions in non-occipital cortex. (**D**) Response time-course plots from the non-illusory conditions (Target Only and Mask Only combined) in occipital versus non-occipital cortex. This analysis controls for the possibility that occipital visual circuits have a higher degree of blood flow than non-occipital circuits. On the contrary, occipital BOLD signal to non-illusory stimuli is relatively low, as compared to non-occipital BOLD signal. Error bars in panels B, C, and D represent SEM between subjects. Reprinted from Tse, et al. (2005).

Having determined the lower boundary in the visual hierarchy for the visibility of simple targets, we set out to determine the upper boundary. To do this, we isolated the parts of the brain that both showed an increase in BOLD signal when the visible stimuli from the non-illusory conditions (Target Only and Mask Only) were displayed, as well as a decrease in BOLD signal when the same targets were rendered less visible by visual masking. Surprisingly, only areas within the occipital lobe showed differential activation between visible and invisible targets (Figure 12).

**Figure 13.**
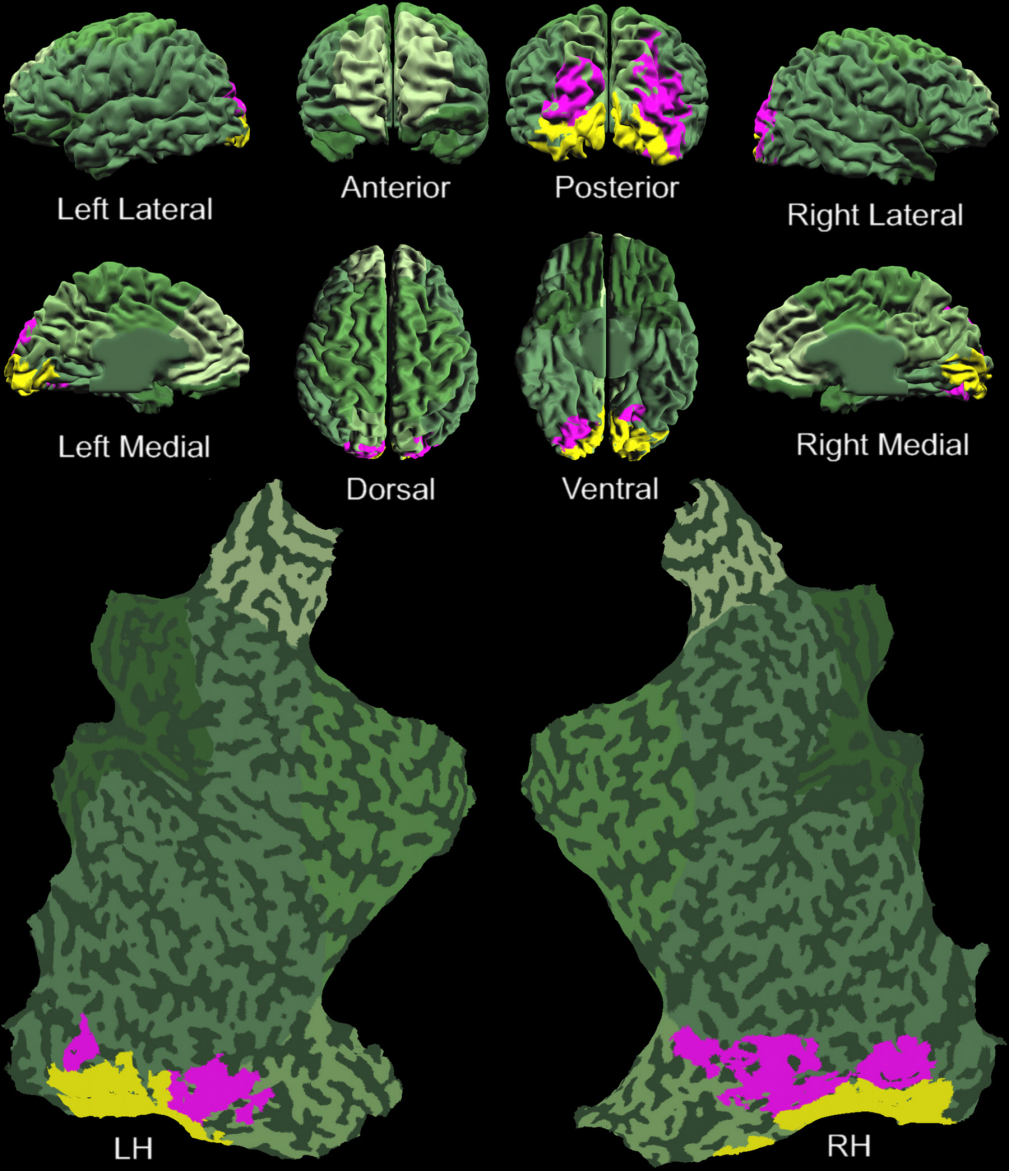
Layout of retinotopic areas that potentially maintain awareness of simple targets. An individual brain model from all perspectives, including both hemispheres flat-mapped, overlaid with the functional activation from one typical subject. The yellow shaded areas are those portions of the brain that did not show significant dichoptic masking (as in Figure 11B & 11C), and thus are ruled out for maintaining visual awareness of simple targets. The pink colored voxels represent the cortical areas that exhibited significant dichoptic masking, and thus are potential candidates for maintaining awareness of simple targets. Reprinted from Tse, et al. (2005).

These combined results suggested that visual areas beyond V2, within the occipital lobe, are responsible for maintaining our awareness of simple unattended targets (Figure 13). Awareness of complex targets is expected to lie outside the occipital lobe, where higher visual processes take place.

In summary, our results show that masking in the early visual system is not caused by feedback from higher cortical areas that also cause dichoptic masking and interocular suppression. It follows that the circuit that causes masking must be ubiquitous enough and simple enough that it exists at many or possibly all levels of the visual system. Lateral inhibition may be such a circuit. Lateral inhibition is the basis for all known receptive field structures in the visual system, and so it must be ubiquitous to all visual areas. This idea is strengthened by our findings that lateral inhibition increases iteratively at each progressive level of the visual hierarchy.

## Verification of the lateral inhibition feedforward model of visual masking

The discussion thus far has reviewed the research for and against the role of feedback in visual masking. The current evidence supports a feedforward model based on lateral inhibition (Herzog et al., 2003; Macknik, 2006; Macknik & Livingstone, 1998; Macknik & Martinez-Conde, 2004b; Tucker & Fitzpatrick, 2006). If this model is correct, one should be able to verify it in a number of independent ways.

One prediction of the model is that luminance increments and decrements should result in neural transients in the primary visual cortex, and that transients should rapidly trigger lateral inhibition. Tucker and Fitzpatrick (2006) have shown, through intracellular recordings in the primary visual cortex, that luminance-evoked transients drive local lateral inhibition.

**Figure 14.**
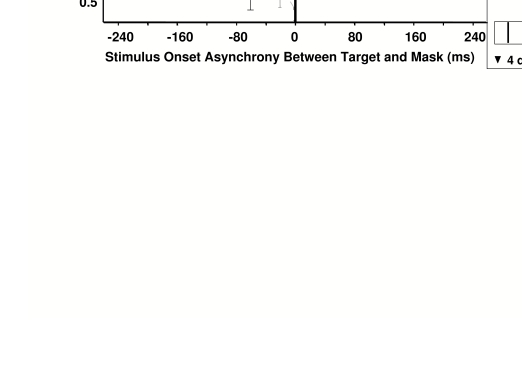
Psychophysical length-discrimination measurements of visual masking from 23 human subjects using overlapping opaque masks of varied size (the distance from the mask’s edge to the target’s edge was 0°, 0.5°, 1°, 2°, or 4° as indicated in the insert). The subject’s task was to fixate on the central black dot and choose the longer target (right or left). Targets were black bars presented for 30 milliseconds; masks were also black and presented for 50 milliseconds. Targets turned on at time 0 ms, and masks were presented at various onset asynchronies so that they came on before, simultaneous to, or after the target in 20 ms steps. Stimulus onset asynchronies (SOAs) to the left of zero indicate forward masking conditions and SOAs greater than zero indicate backward masking. Miniature gray markers with dotted connecting lines represent conditions during which the target and mask overlapped in time and so the target was partially or completely occluded by the mask. The targets were 0.5° wide and had varied heights (5.5°, 5.0°, or 4.5°) and were placed 3° from the fixation dot. The mask was a bar 6° tall with varied widths, spatially overlapped and centered over each target. There were 540 conditions (2 possible choices X 2 differently sized target sets to foil local cue discrimination strategies X 5 overlapping mask sizes X 27 stimulus onset asynchronies). Each condition was presented in random order 5 times to each subject, over a period of 2 days, for a total of 62,100 trials (summed over all 23 subjects). Reprinted from Macknik, et al. (2000).

Another prediction is that transient responses to spatiotemporal edges should be responsible for both target visibility (Macknik & Livingstone, 1998; Macknik et al., 2000), and also the suppressive action of masks (Macknik & Martinez-Conde, 2004a; Macknik et al., 2000). To test whether masks are most inhibitory at their spatial edges, we presented various sized masks that overlapped targets of stable size (Macknik et al., 2000). This experiment was based on designs originally employed by the Crawford, Rushton, and Westheimer groups (Crawford, 1940; Rushton & Westheimer, 1962; Westheimer, 1965, 1967, 1970), but with the innovation that the masks were both varied in size and not presented contemporaneously with the target (Figure 14). As the masks’ edges moved away from the targets’ edges (that is, as the masks grew in size), the strength of the masking decreased. This confirmed that the masks’ spatial edges, as opposed to their interior, evoke the greatest inhibition to target visibility.

**Figure 15.**
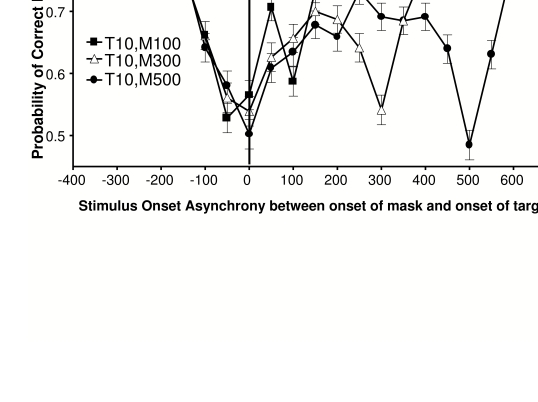
Human psychophysical length-discrimination measurements of visual masking effects from 11 human subjects using non-overlapping masks of varied duration (100, 300, or 500 ms). SOA here represents the period of time between the onset of the mask and the onset of the target (and so it has the opposite meaning than in Figures 3, 4 and 14). Masks (two 6° tall bars with a width of 0.5° flanking each side of each target) appeared at time 0, and targets could appear earlier (backward masking), simultaneously, or later (forward masking), in 50 ms steps. Targets were black and presented for 10 ms duration and masks were flanking black bars that abutted the target. Notice that target visibility is most greatly affected when the masks turn on and off. Reprinted from Macknik, et al. (2000).

To test whether masks were most inhibitory at their temporal edges, we conducted an experiment to determine the times of maximal inhibition during the mask’s lifetime: according to the lateral inhibition feedforward model, these times should be the onset and termination of the mask. We presented a long duration mask and assessed target visibility at various times during the mask’s lifetime (Macknik & Martinez-Conde, 2004a; Macknik et al., 2000) (Figure 15). This experimental design followed from Crawford (Crawford, 1947), but with the important modification that we also varied the duration of the mask. No previous experiment had varied mask duration and so it had not been possible to establish whether inhibitory effects near the termination of the mask were truly caused by the mask’s termination, or whether they were delayed effects of the mask’s onset.

The spatiotemporal lateral inhibition feedforward model of visual masking predicts several visual masking and other illusions, such as the Standing Wave of Invisibility (SWI) illusion, Temporal Fusion, and Flicker Fusion. These are reviewed in detail elsewhere (Macknik, 2006).

Herzog et al. showed that not only first order luminance edges but also second order edges, and in generalany kind of inhomogeneities, are important for masking, and can be mediated by lateral inhibition mechanisms (Herzog & Fahle, 2002; Herzog & Koch, 2001).

### The Standing Wave of Invisibility

**Figure 16.**
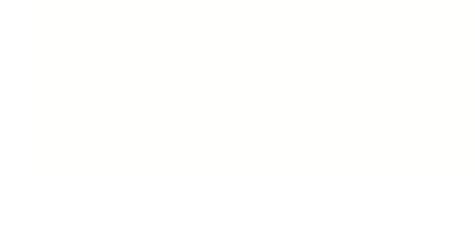
The time-course of events during the Standing Wave of Invisibility illusion (SWI). A flickering target (a bar) of 50 ms duration is preceded and succeeded by two counter-phase flickering masks (two bars that abut and flank the target, but do not overlap it) of 100 ms duration that are presented at the time optimal to both forward and backward mask the target. Reprinted from Macknik (2006).

The SWI illusion was the first perceptual prediction of the spatiotemporal feedforward lateral inhibition model. This illusion combines optimal forward and backward masking in a cyclic fashion, thus suppressing all transient responses associated with each flicker of the target (Figure 16). Without the mask, the target is a highly salient flickering bar, but with the mask present, the target becomes perceptually invisible (Macknik & Haglund, 1999; Macknik & Livingstone, 1998; Macknik & Martinez-Conde, 2004a, 2004b; Macknik et al., 2000; Tse et al., 2005). To the best of our knowledge, this is the first illusion to have been predicted from neurophysiological data, rather than the other way around. The Enns and McGraw groups studied the psychophysics of the SWI illusion (Enns, 2002; McKeefry, Abdelaal, Barrett, & McGraw, 2005).

Breitmeyer and Öğmen (2006) stated that the SWI illusion is the strongest form of visual masking known. However, they credited Werner (Werner, 1935) with the original discovery of the SWI. In doing so they changed the original definition of the SWI illusion. As described above, the SWI illusion (Macknik & Livingstone, 1998) is defined by the combination of optimal forward and backward masking in a single sequence to achieve maximal masking of the target. Breitmeyer and Öğmen redefined the SWI illusion as occurring “when a sequence composed of a target and a surrounding mask is cycled” (Breitmeyer & Öğmen, 2006, pg. 68). However, the most critical feature of the SWI is not the cycling per se, but the combination of optimal forward and backward masking.” (Where “combination of optimal forward and backward masking” is emboldened. Werner (1935) cycled target and mask in either forward or backward masking, but not in both. Moreover, Macknik and Livingstone (1998) first determined the optimal parameters for forward and backward masking: no previous study had varied the duration of both target and mask in order to assess the optimal ISI for forward masking and STA for backward masking. Thus while there may have been a number of cyclic versions of visual masking in the past, the primary innovation of the SWI illusion was not its cyclic nature, but the fact that it first combined optimal forward and backward masking of the same target.

## The functional properties of feedback

We have discussed the data for and against the role of feedback in visual masking, and concluded that there is no strong evidence for feedback. Instead, we have proposed a feedforward model of visual masking based on the same lateral inhibitory circuits that serve to form receptive field structure and to process the spatiotemporal edges of stimuli. However, given that feedback connections exist and make up such a large proportion of the neuroanatomical connectivity, we also concede that feedback must serve an important functional role. Here we review the literature on feedback processes in the visual system, and we propose a role for feedback that may explain the massive number of corticocortical and corticogeniculate back projections.

### Anatomical evidence of feedback within the visual hierarchy

The mammalian visual system includes numerous brain areas that are profusely interconnected. With few exceptions, these connections are reciprocal (Felleman & Van Essen, 1991). In the primate visual system, corticocortical feedforward connections originate mainly in the superficial layers, although they may also arise from the deep layers (less than 10-15% of the connections), and they terminate in layer 4. Feedback connections originate in both superficial and deep layers, and they usually terminate outside of layer 4. In the human visual system, both feedforward and feedback connections can be observed before birth, although feedforward connections reach maturity before feedback connections. At first, both types of connections originate and terminate solely in the deep layers. At 7 weeks of age, both types of fibers reach the superficial layers. At 4 months of age, feedforward connections are fully mature, whereas feedback connections are still at an immature stage (Burkhalter, Bernardo, & Charles, 1993).

Although anatomical feedback connections are ubiquitous throughout the visual cortex, subcortical regions also receive a large amount of feedback from cortical areas. For instance, corticogeniculate input is the largest source of synaptic afferents to the cat LGN. Whereas retinal afferents only encompass 25% of the total number of inputs to LGN interneurons, 37% of the synaptic contacts come from the cortex. In the case of relay cells, the respective percentages are 12% vs. 58% (Montero, 1991). Boyapati and Henry (Boyapati & Henry, 1984) concluded that feedback connections from the cat visual cortex to the LGN concentrated a larger fraction of fine axons than feedforward connections, resulting in comparatively slower conduction speeds. However, Girard and colleagues (Girard, Hupe, & Bullier, 2001) more recently found that feedforward and feedback connections between areas V1 and V2 of the monkey have similarly rapid conduction speeds.

### Physiological evidence for feedback

Most physiological studies in the visual system have found that feedback connections enhance or decrease neuronal responsiveness, without fundamentally altering response specificity. Although the role of such modulation in our visual perception remains unclear, it has been suggested that feedback may be involved in attentional mechanisms (Martinez-Conde et al., 1999).

Corticogeniculate connections to the LGN are retinotopically organized, and they preferentially end on LGN layers with the same ocular dominance as the cortical cells of origin (Murphy & Sillito, 1996). Corticocortical feedback connections are also retinotopically specific (Salin, Girard, Kennedy, & Bullier, 1992). For instance, there is a functional projection from area 18 to area 17 neurons with a similar retinotopic location (Bullier, McCourt, & Henry, 1988; Martinez-Conde et al., 1999; Salin et al., 1992; Salin, Kennedy, & Bullier, 1995).

In the cat visual cortex, electrical stimulation from areas 18 and 19 demonstrated 50% of monosynaptic connections with superficial layers of area 17, in regions with similar functional properties, such as retinotopic location (Bullier et al., 1988). Mignard and Malpeli also found that inactivation of area 18 in the cat led to decreased responses in area 17 (Mignard & Malpeli, 1991). Martinez-Conde et al (1999) found that focal reversible inactivation of area 18 produced suppressed or enhanced visual responses in area 17 neurons with a similar retinotopy. In most area 17 neurons, orientation bandwidths and other functional characteristics remained unaltered, suggesting that feedback from area 18 modulates area 17 responses without fundamentally altering their specificity.

In the squirrel monkey, Sandel and Schiller (1982) found that most area V1 cells decreased their visual responses when area V2 was reversibly cooled, although a few cells became more active (Sandell & Schiller, 1982). Orientation selectivity remained unchanged, although direction selectivity decreased in some instances. Bullier et al. (1996) reported in the cynomologous monkey that, following GABA inactivation of area V2, V1 neurons showed decreased or unchanged responses in the center of the classical receptive field, but increased responses in the region surrounding it (Bullier, Hupe, James, & Girard, 1996). These results were supported by subsequent findings in areas V1, V2 and V3 following area MT inactivation (Hupe et al., 1998). More recently, Angelucci and colleagues (Angelucci & Bressloff, 2006; Angelucci, Levitt, & Lund, 2002) have suggested that area V1 extraclassical receptive field properties arise from area V2 feedback.

In summary, physiological studies as a whole suggest that feedback connections in the visual system may play a modulatory role, rather than a specific role, in shaping the responses of hierarchically lower areas. This evidence agrees with the “no-strong-loops” hypothesis formulated by Crick and Koch (1998b). The no-strong-loops hypothesis proposes that all strong connections in the visual system are of the feedforward type. That is, “*the visual cortex is basically a feedforward system that that is modulated by feedback connections”*, which is “*not to say that such modulation may not be very important for many of its functions*”. Crick and Koch argued that “*although neural nets can be constructed with feedback connections that form loops, they do not work satisfactorily if the excitatory feedback is too strong*”. Similarly, if feedback connections formed “*strong, directed loops*” in the brain, the cortex would as a result “*go into uncontrolled oscillations*”. Therefore, the relative number of feedback vs. feedforward anatomical connections to any given visual area may be misleading as to the respective roles of such connections. For instance, the fact that the cat LGN receives substantially larger numbers of synapses from the cortex than from the retina (Montero, 1991) does not necessarily mean that corticogeniculate connections are more important than retinogeniculate connections in determining the response characteristics of LGN neurons.

### Top-down attention as a unitary explanation for feedback anatomy in the visual system

Based on the above evidence, one important role for feedback may be to carry attentional modulation signals. Other modulatory roles for feedback remain possible, but none are as clearly established. Thus it may be that all of the feedback connectivity exists for the sole purpose of mediating facilitatory and suppressive attentional feedback. At first, given the massive extent of anatomical feedback vs. feedforward connections, this possibility may seem unlikely. Indeed, the great extent of feedback connectivity suggests to some that feedback must have a large number of roles (Sherman & Guillery, 2002; Sillito & Jones, 1996). However, we will argue here that the need for top-down attentional modulation, alone, could potentially explain the great number of feedback connections. Because ascending circuits in the visual system form a primarily hierarchical and labeled-line structure, it follows that feedback inputs must require more wiring than feedforward inputs, to send back even the simplest signal.

To illustrate the logic of this argument, let us consider the anatomical connectivity between the LGN and V1. As previously described, LGN relay cells receive more numerous feedback from the cortex than the feedforward inputs they receive from the retina. However, because cortical receptive fields are orientation selective, and since LGN receptive fields are not oriented themselves, any functionally significant feedback from a given cortical retinotopic location must represent all orientations. That is, for each unoriented geniculocortical feedforward connection, there must be many oriented corticogeniculate feedback connections; each with a different orientation, so that the sum of all feedback inputs may fill the orientation space. Otherwise, if the orientation space of the feedback was not filled completely, LGN receptive fields would show a significant orientation bias. Thus, anatomical feedback connectivity must be large so as to represent the entire orientation space at each retinotopic location. However, because of their orientation selectivity, only a fraction of the feedback connections will be functional at any given time, depending on the orientation of the stimulus, whereas the feedforward connection will be constitutively active irrespective of orientation. In summary, the massive feedback versus feedforward connectivity ratio can be misleading: this large ratio does not necessarily mean that feedback signals are more important or more physiologically relevant than feedforward signals, because higher visual areas are more selective than lower visual areas, and so only a relatively small fraction of the feedback may be expected to be active at any given moment. Rather, feedback connections may need to tile the entire receptive field space of the higher level, or else the feedback would impose high-level receptive field properties on the lower areas. Figure 7 illustrates this idea in terms of dichoptic versus monoptic processing circuits.

Therefore, from basic principles of hierarchical connectivity in the visual system (i.e. ascending pathways become more complex in their receptive field structure as they rise through the brain), we conclude that anatomical feedback connections must be more numerous than feedforward connections. This would be true even if there was just a single functional purpose for feedback.

If we combine these ideas with the Crick and Koch’s no-strong-loops hypothesis, we may conclude that feedback can only be moderately modulatory as compared to feedforward inputs, despite the fact that feedback connections are more numerous. This concept follows from the known physiology: besides their lack of orientation selectivity, another feature that distinguishes LGN from V1 receptive fields is their smaller size (Allman, Miezin, & McGuinness, 1985; Desimone, Schein, Moran, & Ungerleider, 1985; Kastner, Nothdurft, & Pigarev, 1999; Knierim & Van Essen, 1991; Zeki, 1978a, 1978b). If feedback connections from V1 to the LGN were as strong as their feedforward counterparts (in physiological terms) then LGN receptive fields would be as large as V1 receptive fields, but they are not. That is, because LGN receptive fields are smaller than V1 receptive fields, feedback from V1 must be weaker than the input from the retina.

It follows from these ideas that when feedback is operational, some receptive field properties, such as size, which continues to increase throughout the visual hierarchy (Allman et al., 1985; Desimone et al., 1985; Kastner et al., 1999; Knierim & Van Essen, 1991; Zeki, 1978a, 1978b) will be fed back from higher to lower levels. Thus we may predict that, if attention is carried by feedback connections, the earlier receptive fields should get bigger in size when attention is applied actively. This prediction has been confirmed experimentally (He, Cavanagh, & Intriligator, 1996; Williford & Maunsell, 2006).

To conclude, feedback may have no other function than to modulate (facilitate or suppress) feedforward signals as a function of attentional state.

## The role of visual masking, binocular rivalry, attention, and feedback in the study of visual awareness

Let us assume that visual awareness is correlated to brain activity within specialized neural circuits, and that not all brain circuits maintain awareness. It follows that the neural activity that leads to reflexive or involuntary motor action may not correlate with awareness because it does not reside within awareness-causing neural circuits (Macknik & Martinez-Conde, in press).

Let us also propose that there is a “minimal set of conditions” necessary to achieve visibility, in the form of a specific type (or types) of neural activity within a subset of brain circuits. This minimal set of conditions will not be met if the correct circuits have the wrong type of activity (too much activity, too little activity, sustained activity when transient activity is required, etc). Moreover, if the correct type of activity occurs, but solely within circuits that do not maintain awareness, visibility will also fail. Finding the conditions in which visibility fails is critical to the research described here: although we do not yet know what the minimal set of conditions is, we can nevertheless systematically modify potentially important conditions to see if they result in stimulus invisibility. If so, the modified condition will potentially be part of the minimal set.

To establish the minimal set of conditions for visibility we need to answer at least 4 questions (Macknik, 2006). The questions and their (partial) answers, are as follows:

 1) *What stimulus parameters are important to visibility?*

The spatiotemporal edges of stimuli are the most important parameters to stimulus visibility (Macknik et al., 2000).

 2) *What types of neural activity best maintain visibility (transient versus sustained firing, rate codes, bursts of spikes, etc – that is, what is the neural code for visibility)?*

Transient bursts of spikes best maintain visibility (Macknik & Livingstone, 1998; Macknik et al., 2000; Martinez-Conde, Macknik, & Hubel, 2000, 2002).

3) *What brain areas must be active to maintain visibility?*

Visual areas downstream of V2, lying within the occipital lobe, must be active to maintain visibility of simple unattended targets (Macknik & Martinez-Conde, 2004a; Tse et al., 2005).

4) *What specific neural circuits within the relevant brain areas maintain visibility?*

The specific circuits that maintain visibility are presently unknown, but their responsivity is modulated by lateral inhibition (Macknik & Livingstone, 1998; Macknik & Martinez-Conde, 2004a, 2004b; Macknik et al., 2000).

We must also determine the set of standards that will allow us to conclude that any given brain area, or neural circuit within an area, is responsible for generating a conscious experience. Parker and Newsome developed a “*list of idealized criteria that should be fulfilled if we are to claim that some neuron or set of neurons plays a critical role in the generation of a perceptual event*” (Parker & Newsome, 1998). If one replaces the words “perceptual event” with “conscious experience”, Parker and Newsome’s list can be used as an initial foundation for the neurophysiological requirements needed to establish whether any given neuron or brain circuit may be the neural substrate of awareness (Macknik & Martinez-Conde, in press). Parker and Newsome’s list follows:

1) The responses of the neurons and of the perceiving subject should be measured and analyzed in directly comparable ways.

2) The neurons in question should signal relevant information when the organism is carrying out the chosen perceptual task: Thus, the neurons should have discernable features in their firing patterns in response to the different external stimuli that are presented to the observer during the task.

3) Differences in the firing patterns of some set of the candidate neurons to different external stimuli should be sufficiently reliable in a statistical sense to account for, and be reconciled with, the precision of the organism’s responses.

4) Fluctuations in the firing of some set of the candidate neurons to the repeated presentation of identical external stimuli should be predictive of the observer’s judgment on individual stimulus presentations.

5) Direct interference with the firing patterns of some set of the candidate neurons (e.g. by electrical or chemical stimulation) should lead to some form of measurable change in the perceptual responses of the subject at the moment that the relevant external stimulus is delivered.

6) The firing patterns of the neurons in question should not be affected by the particular form of the motor response that the observer uses to indicate his or her percept.

7) Temporary or permanent removal of all or part of the candidate set of neurons should lead to a measurable perceptual deficit, however slight or transient in nature.”

However, visual circuits that may pass muster with Parker and Newsome’s guidelines may nevertheless fail to maintain awareness, as explained below. To guide the search for the neural correlates of consciousness (NCC), some additional standards must be added.

The first additional standard concerns the use of illusions as the tool of choice to test whether a neural tissue may maintain awareness. Visual illusions, by definition, dissociate the subject’s perception of a stimulus from its physical reality. Thus visual illusions are powerful devices in the search for the NCC, as they allow us to distinguish the neural responses to the physical stimulus from the neural responses that correlate to perception. Our brains ultimately construct our perceptual experience, rather than re-construct the physical world (Macknik & Haglund, 1999). Therefore, an awareness-maintaining circuit should express activity that matches the conscious percept, irrespective of whether it matches the physical stimulus. Neurons (circuits, brain areas) that produce neural responses that fail to match the percept provide the most useful information because they can be ruled out, unambiguously, as part of the NCC. As a result, the search for the NCC can be focused to the remaining neural tissue. Conversely, neurons that do correlate with perception are not necessarily critical to awareness, as they may simply play a support role (among other possibilities) without causing awareness themselves.

The second new standard derives from a major contribution of Crick and Koch’s: the distinction between explicit and implicit representations (Crick & Koch, 1998a). In an explicit representation of a stimulus feature, there is a set of neurons that represent that feature without substantial further processing. In an implicit representation, the neuronal responses may account for certain elements of a given feature, however the feature itself is not detected at that level. For instance, all visual information is implicitly encoded in the photoreceptors of the retina. The orientation of a stimulus, however, is not explicitly encoded until area V1, where orientation-selective neurons and functional orientation columns are first found. Crick and Koch propose that there is an explicit representation of every conscious percept.

Here we propose the following corollary to Crick and Koch’s idea of explicit representation: Before one can test a neural tissue for its role in the NCC, such tissue must be shown to explicitly process the test stimulus. This corollary constrains the design of neurophysiological experiments aimed to test the participation of specific neurons, circuits, and brain areas in the NCC.

For instance, if one found that retinal responses do not correlate with auditory awareness, such a discovery would not be carry great weight. The neurons in the eye do not process auditory information, and so it is not appropriate to test their correlation to auditory perception. However, this caveat also applies to more nuanced stimuli. What if V1 was tested for its correlation to the perception of faces versus houses? Faces and houses are visual stimuli, but V1 has never been shown to process faces or houses explicitly, despite the fact that visual information about faces and houses must implicitly be represented in V1. Therefore, one cannot test V1’s correlation to awareness using houses versus faces, and expect to come to any meaningful conclusion about V1’s role in the NCC. Because that form of information is not explicitly processed in V1, it would not be meaningful to the NCC if neurons in V1 failed to modulate their response when the subject is presented with faces versus houses.

It follows that some stimuli are incapable of localizing awareness within specific neural tissues, because no appropriate control exists to test for their explicit representation. For example, binocular rivalry stimuli pose a special problem in the study of visual awareness. Binocular rivalry (Wheatstone, 1838) is a dynamic percept that occurs when two disparate images that cannot be fused stereoscopically are presented dichoptically to the subject (i.e. each image is presented independently to each of the subject’s eyes). The two images (or perhaps the two eyes) appear to compete with each other, and the observer perceives repetitive undulations of the two images, so that only one of them dominates perceptually at any given time (if the images are large enough then binocular rivalry can occur in a piecemeal fashion, so that parts of each image are contemporaneously visible).

Binocular rivalry has been used as a tool to assess the NCC, but has generated controversy because of conflicting results (Macknik & Martinez-Conde, 2004a; Tse et al., 2005). Some human fMRI studies report that BOLD activity in V1 correlates with visual awareness of binocular rivalry percepts (Lee, Blake, & Heeger, 2005; Polonsky, Blake, Braun, & Heeger, 2000; Tong & Engel, 2001). In contrast, other human fMRI studies (Lumer, Friston, & Rees, 1998), and also single-unit recording studies in primates (Leopold & Logothetis, 1996), suggest that activity in area V1 does not correlate with visual awareness of binocular rivalry percepts. One possible reason for this discrepancy is that none of the above studies determined that the visual areas tested contained the interocular suppression circuits necessary to mediate binocular rivalry. That is, since binocular rivalry is a process of interocular suppression, the neural tissue underlying the perception of binocular rivalry must be shown to produce interocular suppression – *explicitly*. Otherwise, it cannot be demonstrated that binocular rivalry is a valid stimulus for testing the NCC in such tissue. Thus, awareness studies using binocular rivalry are valid only in those areas that have been shown to maintain interocular suppression. If binocular rivalry fails to modulate activity within a visual area, one cannot know, by using binocular rivalry alone, if the perceptual modulation failed because awareness is not maintained in that area, or because the area does not have circuits that drive interocular suppression. This is more than just a theoretical possibility: as described earlier, we have shown that the initial binocular neurons of the early visual system (areas V1 and V2) are binocular for excitation, but monocular for inhibition. That is, they fail to process interocular suppression explicitly (Macknik & Martinez-Conde, 2004a; Tse et al., 2005) (Figures Figure 9 and 11).

Since there is no monoptic form of binocular rivalry, one cannot use binocular rivalry by itself to test the strength of interocular suppression. One could use binocular rivalry in tandem with a different stimulus, such as visual masking stimuli, to test for the explicit representation and strength of interocular suppression, as described further below. But in such case, the role of the tissue in maintaining visibility and awareness would have been probed by the visual masking stimuli, thus obviating the need for binocular rivalry stimuli. Because one must rely on non-binocular rivalry stimuli to determine the explicit representation and strength of interocular suppression in a given area, it is not possible to unambiguously interpret the neural correlates of perceptual state using binocular rivalry alone.

Our visual masking studies have shown that binocular neurons in areas V1 (the first stage in the visual hierarchy where information from the two eyes is combined) and V2 of humans and monkeys can integrate excitatory responses between the eyes (Macknik & Martinez-Conde, 2004a; Tse et al., 2005) (Figures 9 and 11). However, these same neurons do not express interocular suppression between the eyes. That is, binocular neurons in V1 are largely binocular for excitation while nevertheless being monocular for suppression. In summary, most early binocular cells do not explicitly process interocular suppression, and so these neurons cannot process binocular rivalry explicitly. Thus binocular rivalry is an inappropriate stimulus to probe early visual areas for the NCC. This result renders the results from binocular rivalry studies that localize visual awareness in the visual system uninterpretable with respect to localizing the NCC: the fact that early visual areas are not correlated to awareness of binocular rivalry is equivalent in significance to concluding that these areas are not correlated to auditory awareness. However, these findings also beg the question of why some studies have concluded that binocular rivalry can occur in low level visual areas (Haynes, Deichmann, & Rees, 2005; Lee et al., 2005; Polonsky et al., 2000; Tong & Engel, 2001; Wunderlich, Schneider, & Kastner, 2005). We propose that the reason for this discrepancy is that these studies have failed to properly control for the effects of attentional feedback, thus confounding apparent inter-ocular suppression effects with attention-modulated activity. Essentially, the subjects in these studies attended to the stimuli of interest, and thus attention itself could be the cause of the retinotopic activation seen in these studies, not inter-ocular inhibition.

Visual masking, on the other hand, has features that make it immune to these shortcomings, and so it is an ideal visual illusion to isolate the NCC. Because visual masking illusions allow us to examine the brain’s response to the same physical target under varying levels of visibility, all we need to do is measure the perceptual and physiological effects of the target when it is visible versus invisible and we will determine many, if not all, of the conditions that cause visibility.

We propose that, to test for explicit processing in neural tissue, one should use a visual illusion, such as visual masking, that can be presented in at least two modes of operation: one mode to ensure that the tissue processes the stimulus explicitly, and one mode to test the correlation to awareness. In visual masking, the monoptic mode establishes that the neural tissue processes masking stimuli explicitly, and then the dichoptic mode can be used to probe the NCC.

The third strategy involves controlling for the effects of attention when designing experiments to isolate the NCC. Attention is a process in which the magnitude of neural activity is either enhanced or suppressed by high-level cognitive mechanisms (Desimone & Duncan, 1995; McAdams & Maunsell, 1999; Moran & Desimone, 1985; Spitzer, Desimone, & Moran, 1988; Williford & Maunsell, 2006). Therefore attention may increase or decrease the likelihood of awareness of a given visual stimulus. However, attention is a distinct process from awareness itself (Merikle, 1980; Merikle & Joordens, 1997; Merikle, Smilek, & Eastwood, 2001). For instance, low-level bottom-up highly salient stimuli (such as flickering lights) can lead to awareness and draw attention, even when the subject is actively attending to some other task, or not attending to anything (i.e. when the subject is asleep). Thus awareness can modulate attention, but the opposite is also true. This double-dissociation suggests that the two processes are mediated by separate brain circuits. It follows that in experiments to isolate the NCC, if the subject is conducting a task that requires attention to the stimulus of interest, then attention and awareness mechanisms may be confounded. Therefore, experiments to isolate the NCC should control for the effects of attention. If experimental manipulation of attentional state affects the magnitude of neural response, then the neural mechanism of interest may not be related to awareness, but instead to attention.

Therefore, we add the following three standards to Parker and Newsome’s list:

18) The candidate neurons should be tested with an illusion that allows dissociation between the physical stimulus and its perception. If the candidate set of neurons is capable of maintaining awareness, the neural responses should match the subjective percept, rather than the objective physical reality of the stimulus.

19) The candidate neurons must explicitly process the type of information or stimulus used to test them.

10) The responses of the neurons, and of the perceiving subject, should be measured with experimental controls for the effect of attention.

## Conclusions

Several models of visual masking require feedback connections to explain the mysterious timing of backward masking. While some physiological reports support the role of feedback in visual masking, we have argued here that none of these studies have controlled appropriately for the effects of attention, which is a well-known top-down effect. In contrast, physiological and psychophysical studies that control for attention support feedforward models of visual masking. The spatiotemporal dynamics of feedforward lateral inhibition circuits within the various levels of the visual hierarchy may explain the many different properties of visual masking, including seemingly high-level cognitive effects.

We have reviewed the literature on the anatomy and physiology of feedback in the visual system and concluded that feedback may exist solely to mediate attentional facilitation and suppression. We have also proposed that the large ratio of feedback to feedforward connections may not indicate a more significant physiological impact of feedback, but it may be a requirement of any feedback mechanism that operates within a hierarchical pathway in which receptive fields go from simple to complex as one rises within the hierarchy.

Finally, we have discussed the strengths of visual masking in the study of visual awareness, as compared to binocular rivalry, and have concluded that visual masking is an ideal paradigm in awareness studies, whereas binocular rivalry has serious shortcomings as a means to localize the NCC. Using visual masking as a tool, we have developed several new standards that must be met to determine the role of a neural circuit in maintaining the NCC.
